# Contribution to the knowledge of the bumblebee fauna of Afghanistan (Hymenoptera, Apidae, *Bombus* Latreille)

**DOI:** 10.3897/zookeys.973.54796

**Published:** 2020-10-05

**Authors:** Guillaume Ghisbain, Paul H. Williams, Denis Michez, Michael G. Branstetter, Pierre Rasmont

**Affiliations:** 1 Laboratory of Zoology, Research Institute of Biosciences, University of Mons (UMONS), Mons, Belgium University of Mons Mons Belgium; 2 Department of Life Sciences, Natural History Museum, Cromwell Road, London SW7 5BD, UK Natural History Museum London United Kingdom; 3 U.S. Department of Agriculture, Agricultural Research Service, Pollinating Insects Research Unit, Utah State University, Logan, Utah 84322, USA Utah State University Logan United States of America

**Keywords:** Asian bees, checklist, Pamir, pollinators, taxonomy

## Abstract

Bumblebees (Hymenoptera: Apidae: genus *Bombus* Latreille, 1802) constitute an important group of pollinators for many wild plants and crops in north temperate regions and South America. Although knowledge of these insects has been increasing over the last decades, some geographic areas remain poorly studied and additions to the knowledge of their faunas are infrequent. Afghanistan is one example of a country that is currently underrepresented in the scientific literature despite its high species diversity. For this study, more than 420 new occurrence records were gathered for 17 bumblebee species belonging to all eight subgenera recorded in the country, including the first record of a species closely related to the *Blongipennis* group. Additionally, the first standardized database for Afghan bees is launched, which we hope will be enriched in the future to allow further assessments of population trends for the bumblebees of Afghanistan. Finally, the previously published species records for the country are discussed considering the most recent taxonomic revisions of the genus and key perspectives are highlighted for further work in this understudied country and neighboring regions.

## Introduction

Bumblebees (Hymenoptera: Apidae: genus *Bombus*) constitute a key group of widespread cold-adapted insects, substantially contributing to ecosystem services around the globe through the pollination of numerous wild plants and agricultural crops (Velthuis and van Doorn 2006; Klein et al. 2007). These large colorful bees, represented by ~ 260 described species worldwide (Williams et al. 1998), form an increasingly popular model group for large-scale studies in the fields of biogeography (Williams et al. 2017), population genetics (Ghisbain et al. 2020), evolutionary biology (Tian et al. 2019) and more worryingly, global change biology (Kerr et al. 2015; Rasmont et al. 2015). Bumblebee populations are indeed undergoing serious regressions worldwide, mostly attributable to anthropogenic disturbance such as habitat destruction or climate change (Williams and Osborne 2009; Cameron et al. 2011; Cameron and Sadd 2020; Rollin et al. 2020).

Although long-term and detailed data of bumblebee species are extensively documented in some parts of the world such as Europe (Rasmont et al. 2015) or North America (Williams et al. 2014, 2019) and allow large-scale meta-analyses on their decline (e.g., Kerr et al. 2015; Sirois-Delisle and Kerr 2018), other areas presently remain poorly represented in scientific publications. Afghanistan is a prime example of such a place. Although Afghanistan is particularly diverse in terms of its ecosystems and therefore its flora and fauna, the country remains poorly represented in the scientific literature on insects, with the only exception being the order Lepidoptera (Howarth and Povolný 1973, 1976; Wyatt and Omoto 1966 and more recently Tshikolovets 2017 and Tshikolovets et al. 2018). For bumblebees, it has been recently suggested that Afghan mountains were likely to have acted as a climatically suitable historical bridge allowing the spread of *Mendacibombus* bumblebees from the high elevations of Central Asia toward the Middle East and Europe (Williams et al. 2016, 2017). The Afghan mountains therefore constitute an interesting area for faunal assessments and studies in the field of biogeography.

After the contributions and faunal reviews of Reinig (1930, 1940), Richards (1951), and Tkalců (1968), no subsequent studies have added consequential new data to the bumblebee fauna of Afghanistan. To our knowledge, no occurrence records of bumblebee specimens have been published from Afghanistan in recent decades due to the obvious reason of prolonged human conflict within its territory. Turbyville et al. (2013) and Dunford et al. (2014) discussed and presented a checklist of Afghan Hymenoptera, including a partial list of the bumblebee fauna, but these lists were only meant to include insects potentially harmful to soldiers during their stay in the country, and cannot therefore be regarded as proper additions to the present knowledge of the Afghan bumblebee fauna.

The latest faunal review to date (Tkalců 1968) recorded 21 bumblebee species for Afghanistan. Taking into account more recent taxonomic revisions and current synonymies, Tkalců’s checklist would now be reduced to 17 valid species. In this paper, we provide new occurrence records for several of those species, considering the currently accepted synonymies of the genus.

## Materials and methods

We studied the personal collections of the entomologists G. Ebert, H. Huss, C. Naumann, and W.F. Reinig (deposited in the University of Mons, UMONS, Belgium) as well as the museum collections of the Natural History Museum (NHMUK, London, England), and the State Museum of Natural History Karlsruhe (Karlsruhe, Germany), gathering *inter alia* specimens from Afghanistan mainly collected in the 1960’s and 1970’s. Identifications of the specimens were performed using reference specimens from the NHMUK as well as previous taxonomic studies of the bumblebee fauna of Afghanistan, the region of Kashmir and the mountain range of Pamir (mainly Reinig 1930; Tkalců 1968 and Williams 1991). All of the information present on the labels of the examined specimens is listed in the results section, sorted by subgenus and species. However, in order to facilitate easy reuse and updates to the bumblebee Afghan data as part of further studies, we gathered and standardized all the available information into a separate database published with the present study (Suppl. material 1). This appendix gathers all label information from examined specimens (location, date, altitude, collector, identifier) and whenever possible includes GPS coordinates to specimens based on the label data and using the World Geodetic System 1984 (WGS84). This was only done if the coordinates were very likely to correspond to original collecting sites. Because of uncertainty in precisely locating some sites, we assigned an error measurement to coordinates from 1 to 100 km. No coordinates were assigned to specimens whose original collecting locality labelling was highly uncertain or could correspond to multiple sites separated by more than 100 km.

Finally, because a few of our specimens belong to the almost morphologically unidentifiable bumblebees from the subgenus *Bombus s*. *str.* (see Williams et al. 2012), we used preliminary phylogenetic results from an ongoing study that aims to resolve problematic taxonomic cases within the genus *Bombus*. Briefly, this molecular approach combines the targeted enrichment of ultra-conserved elements (UCEs) with multiplexed next-generation sequencing (NGS) (Branstetter et al. 2017a, b) and allows for efficient recovery of many nuclear and mitochondrial (e.g., COI) loci from museum-preserved specimens. These data were integrated with previously published phylogenies (Williams et al. 2012) to give insights into the phylogenetic relationships between our examined specimens and what is known from the most up-to-date taxonomic literature.

## Results

### New occurrence records for the Afghan bumblebee fauna

**Figure 1.**
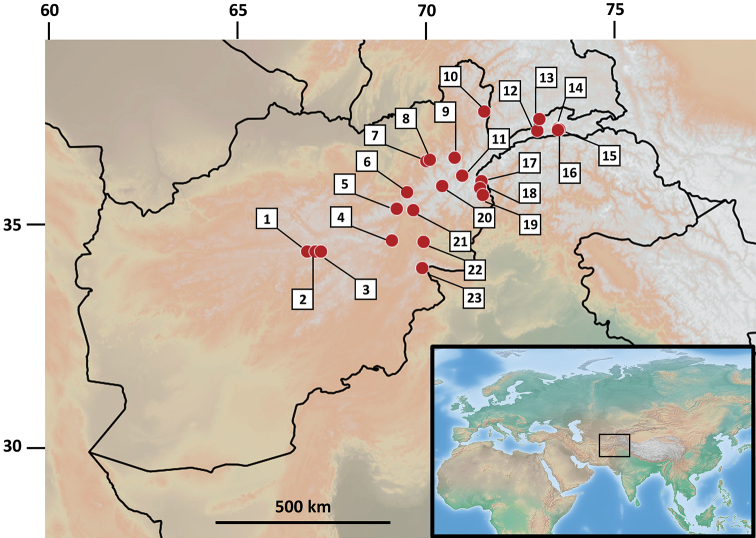
Map of Afghanistan and neighboring regions depicting the sampling examined as part of this study. Numbers correspond to the following locations: 1) Kotal-e-Asgharat; 2) Kotal-e-Narges, D-e-Godar; 3) Ghorghori-e-Panjao, Gaukhana; 4) Pagmangebirge; 5) Salang-Nord, vic. Khindjan / Salang-Paß; 6) Andarab; 7) upper Lezdi valley; 8) Lezdi; 9) Chodja-Mahomed [Kwaja Muhammed]; 10) Shiva-See; 11) Bala Kuran; 12) Kotal-e-Wazir; 13) Darrah-e-Istmotsh 14) Issiktal [Quellflur in Artemisia-Chenopodiensteppe]; 15) Ptukh; 16) Issik; 17) Ahmad Diwana (Baba); 18) Bashgal river; 19) Kamdesh, near Suingal/Shkurigal confluence; 20) Badakshan, Anjuman Pass; 21) Dar.-e-Pandjshir, Kotal-e-Tal; 22) Sarobi; 23) Safed Koh, Kotkai. GPS coordinates and altitude of these sites are given in Table 1 and in the associated database (Suppl. material 1).

The subgenera are classified following the currently accepted phylogenetic relationships of the genus *Bombus* (Cameron et al. 2007; Williams et al. 2008) and species by alphabetical order. The following symbols are used: ♂ = male, ☿ = worker and ♀ = queen. Sampling locations for which GPS coordinates could be assigned are represented by a number in square brackets (see the corresponding name site and associated latitude, longitude and altitude in the Table 1 and their mapping in the Figure 1). Sampling sites that could not be geographically identified with a high degree of certainty are associated with a question mark in square brackets. All the information on the whole sampling is standardized in the database in Suppl. material 1.

**Table 1.** Locations in Afghanistan for which GPS coordinates could be assigned. Latitude (Lat.) and longitude (Long.) are given in decimal degrees and rounded to two decimal places. Site numbers correspond to the locations on the map in Figure 1 (ordered in a clockwise pattern) and to the numbers in square brackets in the Results section. See the associated database (Suppl. material 1) for more details about the samples.

Labelled site name	Site number	Lat. (DD) and Long. (DD)	Approximate elevation (m a.s.l.)
Kotal-e-Asgharat E-Ste	1	34.38N, 66.65E	3200
Kotal-e-Narges, D.-e-Godar	2	34.38N, 66.87E	3150
Ghorghori-e-Panjao, Gaukhana	3	34.38N, 67.02E	2800
Pagmangebirge	4	34.61N, 68.9E	2800
Salang-Nord /Salang-Paß	5	35.31N, 69.04E	2100
Andarab	6	35.67N, 69.32E	4250
Upper Lezdi valley	7	36.33N, 69.83E	2240
Lezdi	8	36.36N, 69.91E	1560
Kwaja Muhammed	9	36.41N, 70.58E	3900
Shiva-See	10	37.39N, 71.36E	3100
Bala Kuran	11	36.02N, 70.77E	3200
Kotal-e-Wazir	12	36.98N, 72.783E	4400
Darrah-e-Istmotsh	13	37.23N, 72.83E	4300
Issiktal	14	37.03N, 73.33E	3500
Ptukh	15	37.01N, 73.37E	4900
Issik	16	37.00N, 73.33E	4200
Ahmad Diwana (Baba)	17	35.91N, 71.3E	2600
Bashgal river	18	35.61N, 71.33E	2900
Kamdesh	19	35.75N, 71.25E	3350
Badakshan, Anjuman Pass	20	35.80N, 70.24E	4200
Dar-e-Pandjshir, Kotal-e-Tal	21	35.27N, 69.47E	3800
Sarobi	22	34.59N, 69.76E	1100
Safed Koh, Kotkai	23	34.01N, 69.71E	2350

#### subgenus Mendacibombus Skorikov, 1914

##### Bombus (Mendacibombus) makarjini

Taxon classificationAnimaliaHymenopteraApidae

Skorikov, 1910

B6F3C79A-117F-5F32-B3A8-0AD1C4D22033

###### Published data.

Williams et al. (2016).

###### Material examined.

Kotal-e-Wazir, 07.viii.71, 4400 m, leg. C. Naumann (1♂) (UMONS) [12].

###### Global distribution.

Palaearctic region.

##### Bombus (Mendacibombus) marussinus

Taxon classificationAnimaliaHymenopteraApidae

Skorikov, 1910

EE234C45-A53B-51B7-9067-D1C7A96751CA

###### Published data.

Reinig 1940; Tkalců 1968; Williams et al. (2016).

###### Material examined.

Badakshan, Anjuman Pass, 12.viii.52 (1♀, 6☿☿), 13.viii.52 (1☿), 4200 m, leg. J. Klapperich (NHMUK) [18]; Hindukusch, Andarab, 4250 m, leg. H. Kotzsch E. Kotzsch (1☿) (NHMUK) [6]; Hindukusch, Chodja-Mahomed [Kwaja Muhammed], 3900 m, leg. H. Kotzsch E. Kotzsch, (1♂) [9]; Hindukusch, Nuksan Pass [? near Chitral, Konar], 3750 m, leg. H. Kotzsch E. Kotzsch (1☿, 1♂) (NHMUK) [on the border with Pakistan, at approximately 36.33N, 71.58E]; Issik, 3500 m, leg. H. Huss (no date for 1☿, 1♂; 18.viii.75: 1☿; 22.viii.75: 1☿; 23.viii.75: 8☿; 24.viii.75: 1☿, 1♂; 25.viii.75: 1☿) (UMONS) [15]; Kotal-e-Wazir, 07.viii.71, 4400 m, leg. C. Naumann (1♀) (UMONS) [12]; Pagmangebirge [Paghman mts], 26.viii.53, 2800 m, leg. J Klapperich, (1☿) (NHMUK) [4]; Hindu Kush, Tarest Mts., high valley, 1.ix.67, 3250 m, leg. D.K. Mardon, 36°20'N, 69°50'E (2☿☿) (NHMUK) [7].

###### Global distribution.

Palaearctic region.

##### Bombus (Mendacibombus) turkestanicus

Taxon classificationAnimaliaHymenopteraApidae

Skorikov, 1910

BE0DA0A9-A45F-5442-818D-E7D4BA22FD94

###### Published data.

Williams et al. (2016).

###### Material examined.

Hindu Kush, upper Lezdi valley, 21.viii.67, 2240 m, leg. D.K. Mardon, 36°20'N, 69°50'E (1☿) (NHMUK) [7]; Shiva-See, 2–5.viii.71, 3100 m, D. Müting (2☿☿, 1♂) (NHMUK) [10]; Hindu Kush, Tarest Mts., high valley, 1.ix.67, 3250 m, leg. D.K. Mardon, 36°20'N, 69°50'E (1☿) (NHMUK) [7]; Issik, 4200 m, leg. H. Huss (1☿) (NHMUK) [15].

###### Global distribution.

Palaearctic region.

#### subgenus Subterraneobombus Vogt, 1911

##### Bombus (Subterraneobombus) melanurus

Taxon classificationAnimaliaHymenopteraApidae

Lepeletier, 1835

39815E93-52B9-50DD-AC93-1442812BC396

Figure 2

###### Published data.

Reinig 1940; Richards 1951; Tkalců 1968.

###### Material examined.

C-Afghanistan, Prov. Ghor, Kotal-e-Asgharat E-Ste., 9.vii.1976, 3200 m, leg. C. Naumann (6☿☿) (UMONS) [1]; C-Afghanistan, Prov. Ghor, Kotal-e-Narges, West-Ste; D.-e-Godar, 09.vii.76, 3100–3200 m, leg. C. Naumann (1☿) (UMONS) [2]; E-Afghanistan, Dar-e-Pandjshir, Kotal-e-tal, 30.vii.73, 3800 m, M. N. Khoram (1♀, 3♂♂) (UMONS) [19]; Ghilzai, 05.viii.48, 1780 m, N. Haarlov (3♂♂) (NHMUK) [?]; Grosser Pamir, Issiktal, Frostbodenflur, 24.viii.75, 4100–4350 m, leg. H. Huss, (1♂) (UMONS) [possibly close to 13]; Grosser Pamir, Issiktal, Quellflur in Artemisia-Chenopodiensteppe, 16.viii.75 (1♀, 4☿☿), 17.viii.75 (1☿), 22.viii.75 (3☿☿, 6♂♂), 23.viii.75 (11☿☿, 2♂♂), 25.viii.75 (1♀, 5☿☿, 3♂♂), 3500 m, leg. H. Huss, 37°02'N, 73°20'E (UMONS) [13]; Grosser Pamir, Ptukh, 30.vii.75, leg. H. Huss (1♀) (UMONS) [14]; Hazaradjat, Koh-i-Baba, Pandjao-Umg., 26.vii.61, 2500 m, leg. G. Ebert (1☿) (UMONS) [reference coordinates of the Koh-i-Baba 34.64N, 67.62E]; Hazaradjat, Koh-i-Baba, Shah-tu-Kotal, 20–21.vi.1961, 4000m, leg. G. Ebert (1♀, 2☿☿) (UMONS) [reference coordinates of the Koh-i-Baba 34.64N, 67.62E]; Hindu Kush, 06.viii.68, 2290 m, leg. M. Tong (1☿) (NHMUK) [sampling site not possible to locate, given that the Hindu Kush is – 800 km long]; Hindu Kush, nr Kamdesh confluence of R. Suingal and R. Shkurigal, viii.1977, 11000 ft., P.H. Ryley, 35°45'N, 71°15'E (1♀) (NHMUK) [17]; Tarest Mts., 01.ix.67, 3250 m, leg. D.K. Mardon (1☿); Z-Afghanistan, Koh-i-Baba, S-Seite, Shah-tu-Pass, 17–19.vii.1966, 3000 m, leg. G. Ebert (1♀, 2☿☿, 5♂♂) (UMONS) [reference coordinates of the Koh-i-Baba 34.64N, 67.62E].

**Table 2.** Species examined as part of the present study (✓ = taxon examined in the present study; X = taxon reported in Afghanistan but not examined).

Subgenus(following Williams et al. 2008)	Taxon (according to the latest revisions of Tkalců 1968, Williams et al. 2016, and the present work)	Taxonomic status according to the types examined by PH Williams (https://www.nhm.ac.uk/research-curation/research/projects/bombus/)	Present study
*Mendacibombus*	*afghanus*	Junior synonym of *B.marussinus*	
	*makarjini*	Valid	✓
	*marussinus*	Valid	✓
	*turkestanicus*	Valid	✓
*Subterraneobombus*	*melanurus*	Valid	✓
	*subdistinctus*	Junior synonym of *B.melanurus*	
*Psithyrus*	*branickii*	Valid	✓
	*ferganicus*	Valid	✓
	*morawitzianus*	Valid	X
*Pyrobombus*	*biroi*	Valid	✓
	*kotzschi*	Valid	✓
	*subtypicus*	Valid	✓
*Bombus* *s. str.*	*lucorum jacobsoni*	*Bjacobsoni*	X
	*tunicatus*	Valid	✓
	aff.longipennis	Uncertain taxonomic status	✓
*Melanobombus*	*keriensis*	Valid	✓
	*incertoides*	Valid	X
	*semenovianus*	Valid	✓
*Sibiricobombus*	*asiaticus*	Referred to here as the accepted taxon *B.asiaticuss. l.*	✓
	*miniatocaudatus*		
	*longiceps*		
	*morawitzi*	Valid	✓
	*obtusus* (sspp. *badakshanensis* and *obtusus*)	Valid	✓
*Cullumanobombus*	*cullumanus serrisquama*	Valid	✓

###### Global distribution.

Palaearctic and Oriental regions.

**Figure 2.**
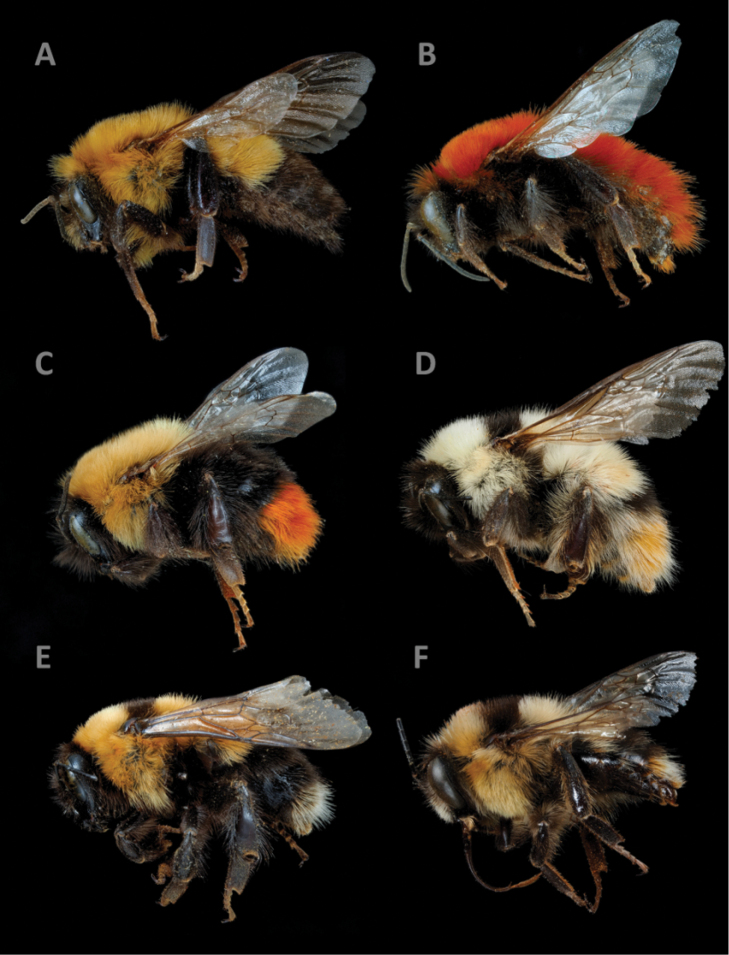
Some of the Afghan bumblebee taxa examined as part of this study. **A**Bombus (Subterraneobombus) melanurus**B**B. (Sibiricobombus) morawitzi**C**B. (Melanobombus) semenovianus**D**B. (Melanobombus) keriensis*s. s.***E**B. (Sibiricobombus) obtusus**F**B. (Sibiricobombus) asiaticus. Photograph credits P. Rasmont.

#### subgenus Psithyrus Lepeletier, 1832

##### Bombus (Psithyrus) branickii

Taxon classificationAnimaliaHymenopteraApidae

(Radoszkowski, 1893)

C1E7123C-5DEC-5D9A-AA29-6A9675320882

###### Published data.

Reinig 1940; Tkalců 1968.

###### Material examined.

Hindu Kush, Tarest Mts., high valley, 29.viii–3.ix.67, 3250 m, leg. D.K. Mardon, 36°20'N, 69°50'E (2♂♂) (NHMUK) [7]; Shiva-See, 2–5.viii.71, 3100 m, D. Müting (4♂♂) (NHMUK) [10].

###### Global distribution.

Palaearctic region and Oriental regions.

##### Bombus (Psithyrus) ferganicus

Taxon classificationAnimaliaHymenopteraApidae

(Radoszkowski, 1893)

4AC08AE5-118C-5BB5-89A6-BCFB226B4209

###### Published data.

Tkalců 1968.

###### Material examined.

Peniger, R. Bashgal, 4–5.viii.65, 9500 ft., leg. G.W. Johnstone (1♀) [other name: Landay-Sin River, 35.61N, 71.33E].

###### Global distribution.

Palaearctic and Oriental regions.

#### subgenus Pyrobombus Dalla Torre, 1880

##### Bombus (Pyrobombus) biroi

Taxon classificationAnimaliaHymenopteraApidae

Vogt, 1911

51ABE20E-EBC5-5FA9-BBE5-AAAE8302D508

###### Published data.

Reinig 1940; Tkalců 1968.

###### Material examined.

Grosser Pamir, Issiktal, Quellflur in Artemisia-Chenopodiensteppe, 23.viii.75, 3500 m, leg. H. Huss, 37°02'N, 73°20'E (1☿) (UMONS) [13]; Shiva-See, 05.viii.71, 3100 m, D. Müting (1☿) (NHMUK) [10].

###### Global distribution.

Palaearctic and Oriental regions.

##### Bombus (Pyrobombus) kotzschi

Taxon classificationAnimaliaHymenopteraApidae

Reinig, 1940

9A2239E8-868F-5D31-9A83-0D7040498DDB

###### Published data.

Reinig 1940; Richards 1951; Tkalců 1968.

###### Material examined.

Hindu Kush, Tarest Mts., high valley, 29.viii–3.ix.67, 3250 m, leg. D.K. Mardon, 36°20'N, 69°50'E (1♂, 1☿) (NHMUK) [7].

###### Global distribution.

Palaearctic region and Oriental regions.

##### Bombus (Pyrobombus) subtypicus

Taxon classificationAnimaliaHymenopteraApidae

(Skorikov, 1914)

3BBD9D17-ED05-5B23-9BF4-86346B7A32AB

###### Published data.

Tkalců 1968.

###### Material examined.

3. Danske Exp. Til Centralasien, [manuscript: St.108], ST. [manuscript] Ghilzai, 05.viii.48 (2♂♂), 11.viii.48 in Surfa? (1♂), N. Haarlov (NHMUK) [?]; Ahmad Diwana (Baba), R. Bashgal Valley, 3.viii.1965, 8500 ft., leg. G.W. Johnstone (2♀, 9☿☿) (NHMUK) [16]; E-Afghanistan, Dar-e-Pandjshir, Kotal-e-tal, 30.vii.73, 3800 m, M. N. Khoram (1♂) (UMONS) [19]; Grosser Pamir, Darrah-e-Istmotsh (nördl. Zweig), südl. Seitental, 05.viii.71, 4200–4400 m, leg. Ebert and Naumann (1♀) (UMONS) [Darya-e Istmotsh: 37.23N, 72.83E]; Hindu Kush, 10.viii.68, 9300 ft., leg. M. Tong (3☿☿); Hindu Kush, nr Kamdesh confluence of R. Suingal and R. Shkurigal, viii.1977, 11000 ft., P.H. Ryley, 35°45'N, 71°15'E (1♂) (NHMUK) [17]; Hindu Kush, Rocky gorge above Lezdi, 17–19.viii.67, 1800–1920 m, leg. D.K. Mardon , 36°20'N, 69°50'E (1♂, 1♀, 7☿☿) (NHMUK) [8]; Hindu Kush, upper Lezdi valley, 21.viii.67, 2240 m, leg. D.K. Mardon, 36°20'N, 69°50'E (1☿) (NHMUK) [7]; NO-Afghanistan, Badachschan, Bala Kuran, 26.vii.1961, 3200 m, leg. G. Ebert (2☿☿) (UMONS) [11]; O-Afghanistan, Sarobi, 1.viii.61, 1100 m, leg. G. Ebert (1☿) (UMONS) [20]; Peniger, R. Bashgal, 4–5.viii.1965, 9500 ft., leg. G.W. Johnstone (2♀♀, 1☿) (NHMUK) [other name: Landay-Sin River, 35.61N, 71.33E]; SO-Afghanistan, Safed Koh, S-Seite, Kotkai, 19–23.6.1966, 2350 m (1☿) (UMONS) [21].

###### Global distribution.

Palaearctic and Oriental regions.

#### subgenus Bombussensustricto Latreille, 1802

##### Bombus (Bombus) aff.longipennis

Taxon classificationAnimaliaHymenopteraApidae

Friese, 1918

FF633360-BC45-53C1-8F58-19F515DC4F70

###### Notes.

New record for Afghanistan.

###### Material examined.

Afghanistan, Grosser Pamir, Issiktal, Quellflur in Artemisia-Chenoponiensteppe, 3500 m, 23.viii.1975 (3☿☿); 22.viii.1975 (4☿☿); 17.viii.1975 (1♂) (UMONS) [13].

###### Global distribution.

Palaearctic and Oriental regions.

##### Bombus (Bombus) tunicatus

Taxon classificationAnimaliaHymenopteraApidae

Smith, 1852

E7102D4B-8F8F-55B2-9E00-32E267EEEEB8

###### Published data.

Tkalců 1968.

###### Material examined.

SO-Afghanistan, Prov. Pastia, Safed Koh, S-Seite, Kotkai, 2350 m, 16–17.vi.1971, rec. Ebert and Naumann, (3♀♀) (UMONS) [21]; SO-Afghanistan, Safed-Koh, S-Seite, Kotkai, 2350m, 14–23.vi.1966 (1♀) (UMONS) [21].

###### Global distribution.

Palaearctic and Oriental regions.

#### subgenus Melanobombus Dalla Torre, 1880

##### Bombus (Melanobombus) keriensis

Taxon classificationAnimaliaHymenopteraApidae

s. s. Morawitz, 1887

099C170A-84C8-55E7-83DA-30A7826EB188

Figure 2

###### Published data.

Reinig 1940; Richards 1951; Tkalců 1968.

###### Material examined.

Hindu Kush, Tarest Mts., high valley, 29.viii–3.ix.67, 3250 m, leg. D.K. Mardon, 36°20'N, 69°50'E (4☿☿) (NHMUK) [7]; 3. Danske Exp. Til Centralasien, [manuscript: St.108], ST. [manuscript] Ghilzai, 05.viii.48 (3☿☿) (NHMUK) [?]; Grosser Pamir Issiktal, 1975–08 (1♀, 4☿☿), 12.viii.75 (at 4200 m, 1☿), leg. H. Huss (UMONS) [possibly close to 13]; Grosser Pamir Issiktal, Salix-bestande, 12.viii.75, 3600 m, leg. H. Huss (3☿☿) (NHMUK) [possibly close to 13]; Grosser Pamir, Darrah-e-Istmotsh (nördl. Zweig), südl. Seitental, 05.viii.71, 4200–4400 m, leg. Ebert and Naumann (2♀♀) (UMONS) [Darya-e Istmotsh: 37.23N, 72.83E]; Grosser Pamir, Issiktal, Frostbodenflur, 24.viii.75, 4100–4350 m, leg. H. Huss, (1☿) (UMONS) [possibly close to 13]; Grosser Pamir, Issiktal, Quellflur in Artemisia-Chenopodiensteppe, 25.viii.75 (42☿☿), 23.viii.75 (10☿☿), 22.viii.75 (7☿☿); 17.viii.75 (5☿☿), 16.viii.75 (2☿☿), 09.viii.75 (2☿☿), 3500 m, leg. H. Huss, 37°02'N, 73°20'E (UMONS) [13]; Hindu Kush, 3.viii.68, 10500 ft., leg. M. Tong (1☿) (NHMUK) [sampling site not possible to locate, given that the Hindu Kush is ~ 800 km long]; Hindu Kush, 6.viii.68, 11500 ft., leg. M. Tong (5☿☿) (NHMUK) [sampling site not possible to locate, given that the Hindu Kush is ~ 800 km long]; N-Afghanistan, Prov. Badakhshan, Grosser Pamir, Kotal-e-Wazir, 4400m, leg. C. Naumann (4☿☿), 07.viii.71 (2♀♀, 6☿☿, 2♂♂) (UMONS) [12]; Salang-Paß, Hindikusch, 13.vii.69, 3500 m, D. Müting (1☿, 2♂♂) (UMONS) [5]; Z-Afghanistan, Koh-i-Baba, S-Seite, Shah-tu-Pass, 17–19.vii.1966, 3000m, leg. G. Ebert (2☿☿) (UMONS) [reference coordinates of the Koh-i-Baba 34.64N, 67.62E].

###### Global distribution.

Palaearctic and Oriental regions.

##### Bombus (Melanobombus) semenovianus

Taxon classificationAnimaliaHymenopteraApidae

(Skorikov, 1914)

E92D5A73-16F6-5FC6-9465-32B885F753F6

Figure 2

###### Published data.

Reinig 1940; Richards 1951; Tkalců 1968.

###### Material examined.

Hindu Kush, Tarest Mts., high valley, 29.viii–3.ix.67, 3250m, leg. D.K. Mardon, 36°20'N, 69°50'E (9☿☿) (NHMUK) [7]; Hindu Kush, nr Kamdesh confluence of R. Suingal and R. Shkurigal, viii.1977, 11000 ft., P.H. Ryley, 35°45'N, 71°15'E (2☿☿) (NHMUK) [17]; Hindu Kush, Rocky gorge above Lezdi, 17–19.viii.67, 1800–1920 m, leg. D.K. Mardon, 36°20'N, 69°50'E (18☿☿) (NHMUK) [7]; Hindu Kush, upper Lezdi valley, 21.viii.67, 2240 m, leg. D.K. Mardon, 36°20'N, 69°50'E (6☿☿) (NHMUK) [7]; Hindu Kush, Lezdi, 15–16.viii.67, 1560 m, leg. D.K. Mardon, 36°20'N, 69°50'E (10☿☿) (NHMUK) [8]; 3. Danske Exp. Til Centralasien, [manuscript: St.126], ST. [manuscript] Marak [?]; 16.viii.48, N. Haarlov (2☿☿) (NHMUK) [?]; 3. Danske Exp. Til Centralasien, [manuscript: St.118], ST. [manuscript] Surfa?, 11.viii.48, N. Haarlov (1☿) (NHMUK) [?]; 3. Danske Exp. Til Centralasien, [manuscript: St.124], ST. [manuscript] Marak, 14.viii.48, N. Haarlov (1☿) (NHMUK) [?]; Hindu Kush, 4.viii.68, 8500 ft., leg. M. Tong (1☿) (NHMUK) [sampling site not possible to locate, given that the Hindu Kush is ~ 800 km long]; Hindu Kush, 6.viii.68, 11500 ft., leg. M. Tong (2☿☿) (NHMUK) [sampling site not possible to locate, given that the Hindu Kush is ~ 800 km long]; Hindu Kush, 3.viii.68, 10500 ft. (19☿☿) (NHMUK) [?]; E-Afghanistan, Salang-Nord, 2100 m, vic. Khindjan, 13.vi.70, 2100 m, leg. C. Naumann (1☿) (UMONS) [5]; Salang-Paß, Hindikusch, 13.vii.69, 3500 m, D. Müting (4☿☿, 2♂♂) (UMONS) [5]; Afghanistan, Salang-Pafs, Nordseite, 17.vi.66, 2650 m, K. Ornoto (1♀) (UMONS) [5]; SO-Afghanistan, Safed Koh, S-Seite, Kotkai, 19–23.vi.1966 (1☿) (UMONS) [21].

###### Global distribution.

Palaearctic region.

#### subgenus Sibiricobombus Vogt, 1911

##### Bombus (Sibiricobombus) asiaticus

Taxon classificationAnimaliaHymenopteraApidae

Morawitz, 1875 sensu lato

6FEB350D-EB64-5CC7-AE07-B37BE2964A52

Figure 2

###### Published data.

Reinig 1940; Richards 1951; Tkalců 1968.

###### Material examined.

3. Danske Exp. Til Centralasien, [manuscript: St.108], ST. [manuscript] Ghilzai, 05.viii.48, N. Harloov (1♀, 2☿☿, 1♂) (NHMUK) [?]; 3. Danske Exp. Til Centralasien, Puistagoli, ST. [manuscript] 106, 02.viii.48, N. Harloov (1♂) (NHMUK) [?]; Ahmad Diwana (Baba), R. Bashgal Valley, 3.viii.1965, 8500 ft., leg. G.W. Johnstone (1♀, 1☿) (NHMUK) [16]; C-Afghanistan, Prov. Bamian, Ghorghori-e-Panjao, Gaukhana, 11.vii.76, 2800 m, leg. C. Naumann (1☿) (UMONS) [3]; C-Afghanistan, Prov. Ghor, Kotal-e-Narges, West-Ste; D.-e-Godar, 09.vii.76, 3100–3200 m, leg. C. Naumann (1♀, 3☿☿) (UMONS) [2]; Grosser Pamir, Darrah-e-Istmotsh (nördl. Zweig), südl. Seitental, 05.viii.71, 4200–4400 m, leg. Ebert and Naumann (1♂) (UMONS) [Darya-e Istmotsh: 37.23N, 72.83E]; Afghanistan, Grosser Pamir, Issiktal, Quellflur in Artemisia-Chenoponiensteppe, 3500m, 22.viii.1975 (1☿), 25.viii.1975, leg. H. Huss (1♀, 2☿☿, 1♂) (UMONS) [13]; Hindu Kush, 8.viii.68, 9500 ft. (1♂), 3.viii.68 (6☿☿; 4♂♂), 6.viii.68 (1♀), leg. M. Tong (NHMUK) [sampling site not possible to locate, given that the Hindu Kush is ~ 800 km long]; Hindu Kush, Rocky gorge above Lezdi, 17–19.viii.67, 1800–1920 m, leg. D.K. Mardon, 36°20'N, 69°50'E (2☿☿, 3♂♂) (NHMUK) [8]; Hindu Kush, Tarest Mts., high valley, 29.viii–3.ix.67, 3250 m, leg. D.K. Mardon, 36°20'N, 69°50'E (1☿) (NHMUK) [7]; Hindu Kush, upper Lezdi valley, 21.viii.67, 2240 m, leg. D.K. Mardon, 36°20'N, 69°50'E (1♂) (NHMUK) [7]; Kl. Pamir, Seitental südl. W-Ende des Kol-e-Tshagmagtin, 20.vii.1971, 4200–4400 m, leg. Ebert and Naumann (1☿) (UMONS) [?]; O-Afghanistan, Salang-Paß, N-Seite (Khinjan), 5–11.vii.1966, 2200 m, leg. G. Ebert (1♂) (UMONS) [5]; SO-Afghanistan, Prov. Paktia, Safed Koh, S-Seite, Kotkai, 16/17.6.1971, 2350 m, leg. Ebert and Naumann (2☿☿) (UMONS) [21]; SO-Afghanistan, Safed Koh, S-Seite, Kotkai, 19–23.6.1966, 2350 m (1☿) (UMONS) [21]; Z-Afghanistan, Koh-i-Baba, S-Seite, Shah-tu-Pass, 17–19.vii.1966, 3000 m, leg. G. Ebert (1☿, 1♂) (UMONS) [reference coordinates of the Koh-i-Baba 34.64N, 67.62E].

###### Global distribution.

Palaearctic and Oriental regions.

##### Bombus (Sibiricobombus) morawitzi

Taxon classificationAnimaliaHymenopteraApidae

Radoszkowski, 1876

DE70A6AE-B36E-59E6-AEE8-B2C5CEEE9B9C

Figure 2

###### Published data.

Reinig 1940; Tkalců 1968.

###### Material examined.

Grosser Pamir, Issiktal, Frostbodenflur, 24.viii.75, 4100 m, leg. H. Huss, 37°02'N, 73°20'E (2♂♂) (UMONS) [13]; Wakhan-Tal, Kotal-e-Dalez, W-Seite, 09.vii.71, 3200–3400 m, leg. Ebert and Naumann (1☿) (UMONS) [around the Wakhan corridor in the NE of Afghanistan: 37.09N, 73.63E].

###### Global distribution.

Palaearctic region.

##### Bombus (Sibiricobombus) obtusus

Taxon classificationAnimaliaHymenopteraApidae

Richards, 1951

2CF9553D-8E82-53C3-9AF4-282BA353F19A

Figure 2

###### Published data.

Richards 1951; Tkalců 1968.

###### Material examined.

Hindu Kush, 10.viii.68 at 9300 ft. (1♂, 2☿☿), 8.viii.68 at 9500 ft. (2☿☿), leg. M. Tong (NHMUK) [sampling site not possible to locate, given that the Hindu Kush is ~ 800 km long]; 3. Danske Exp. Til Centralasien, [manuscript: St.108], ST. [manuscript] Ghilzai, 05.viii.48, N. Haarlov (1☿) [?]; 3. Danske Exp. Til Centralasien, [manuscript: St.126], ST. [manuscript] Marak ; 16.viii.48, N. Haarlov (3☿☿) (NHMUK) [?]; 3. Danske Exp. Til Centralasien, [manuscript: St.124], ST. [manuscript] Marak, 14.viii.48, N. Haarlov (2♂♂) (NHMUK) [?]; C-Afghanistan, Prov. Ghor, Kotal-e-Narges, West-Ste; D.-e-Godar, 09.vii.76, 3100–3200 m, leg. C. Naumann (4♀♀) (UMONS) [2]; Afghanistan Centr., Prov. Bamian, Koh-e-Shorakarak, vic. Samadi, 12.vii.76, 3200 m, leg. C. Naumann (1☿) (UMONS) [reference coordinates of Kōh-e Shōrah Kharak: 34.72N, 67.09E].

###### Global distribution.

Palaearctic region.

#### subgenus Cullumanobombus Vogt, 1911

##### Bombus (Cullumanobombus) cullumanus

Taxon classificationAnimaliaHymenopteraApidae

(Kirby, 1802)

A43C2B31-5FF0-5BBF-BE36-184F7355E5A9

###### Published data.

Richards 1951; Tkalců 1968.

###### Material examined.

C-Afghanistan, Prov. Bamian, Ghorghori-e-Panjao, Gaukhana, 11.vii.76, 2800 m, leg. C. Naumann (1♀) (UMONS) [3]; Hazaradjat, Koh-i-Baba, Shah-tu-Kotal, 20–21.vi.1961, 4000 m, leg. G. Ebert (1♀) (UMONS) [reference coordinates of the Koh-i-Baba 34.64N, 67.62E]; Z-Afghanistan, Koh-i-Baba, S-Seite, Shah-tu-Pass, 17–19.vii.1966, 3000 m, leg. G. Ebert (1♀, 4☿☿) (UMONS) [reference coordinates of the Koh-i-Baba 34.64N, 67.62E].

###### Global distribution.

Palaearctic region.

## Discussion

While the bumblebee fauna of Western and Central Asia has received substantial attention within the last several decades, most notably in the Middle-East (Rasmont and Flagothier 1996; Özbek 1997, 1998, 2000; Monfared et al. 2008, 2009; Boustani et al. 2020) and in the Himalaya (Williams 1991; Williams et al. 2010; Streinzer et al. 2019), Afghanistan however has remained under-studied due to the effects of long-standing effect of human conflict on scientific work in the country.

Most identified specimens in the present work were collected at high elevation in the Afghan Pamir (Wakhan Corridor) and Hindu-Kush mountains (Fig. 1). Bumblebees are indeed regarded as species adapted to cool climates and are especially diversified in montane areas (Williams 1991; Williams et al. 2010; Iserbyt and Rasmont 2012; Rasmont et al. 2015). This habitat preference is explained by the species of the genus being able to (i) thermoregulate efficiently in cold environments (Heinrich 1979), (ii) utilize thermally insulted underground nests built by other inhabitants (e.g., small mammals) and (iii) overwinter with very low food requirements. The Pamir mountain range offers a very hospitable habitat for bumblebees, providing suitable habitat and host plants, including long-corolla flowering plants such as Fabaceae, Scrophulariaceae and Boraginaceae, which have been shown to be attractive for the indigenous *Bombus* species (Reinig, 1930). In the same study, the latter author highlights the short summer period suitable for bumblebees in the Pamir region, from July at 4000 m to September in the Kara Kul Lake (Tadjik Pamir). These observations are congruent with ours, with most specimens being recorded in the month of August. Williams (1991) makes parallel comments for Kashmir, on the other side of the northeastern Afghanistan panhandle, where a short annual season above the freezing point constraints the time available for bumblebee colony development.

Moreover, Reinig (1930) underscores the rarity of the specimens from the subgenus Psithyrus, recording only three specimens of the 1,350 that he caught in the Russian-German expedition he joined in 1928. Our records therefore provide interesting data to improve understanding of the phenology of these rare bumblebees, whose inquiline-host associations can sometimes be speculative or rely on uncertain, old records (Williams 2008; Lhomme and Hines 2019). One of the cuckoo bumblebee species recorded here, *B.branickii*, is suggested to be a social parasite of B. (Melanobombus) keriensis (Williams et al. 1991, 2009). Due to the geographical proximity of the collecting sites of both *Bbanickii* and *B.keriensis*, we provide additional evidence of a probable host-inquiline association of the two species.

Gupta (2004) reports *Bterrestris* (based on one female only) and *Blucorum* as occurring in Afghanistan. However, the author treated the morphologically similar species *Bjacobsoni* as a synonym of *Blucorum*, whereas they are now considered to be distinct species (Williams et al. 2012). Separating species in the *Bterrestris* complex based on morphology and color pattern is an arduous task, likely to be unreliable in most cases, especially for workers or discolored males (Rasmont 1984; Carolan et al. 2012; Williams et al. 2012). In the Pamir range, many taxa of this group have been reported: *Blucorumalaiensis* (described in Reinig 1930), *Blucorum* (reported in Reinig 1940) and *Blucorumjacobsoni* (reported by Tkalců 1968). The latter taxon, *jacobsoni* Skorikov (1912), is now strongly supported to represent a separate species and is presently reported as endemic to Kashmir (Williams et al. 2012). The status of the taxon *alaiensis*Reinig (1930) remains unclear but could be a synonym of *lucorum* (Williams et al. 2012). Regarding our specimens that appear very similar to *Blucorumsensu lato*, preliminary DNA sequencing efforts with the UCE approach (to be presented in a later study), indicate that the present individuals are more closely related to the *Blongipennis* species complex than *Blucorum*. We therefore have decided to assign the name B (Bombus) aff. longipennis to these specimens until further work (e.g., in neighboring regions) can clarify the situation.

Although we did not examine all of the bumblebee collections of Tkalců, Reinig, or Richards, the previous records of B (Melanobombus) incertoides could correspond to specimens of *Bkeriensis s*. *str.*, according to the ongoing global revision of *Melanobombus* (Williams et al. in prep) that suggests that the taxon *Bincertoides* is only present in Mongolia.

Studies addressing the taxonomic relationships of the examined taxa will constitute an essential starting point for further revisions of the Afghan fauna. Highly polymorphic species complexes such as *B.asiaticus* remain enigmatic due to morphological convergence, and many others have not even been collected recently enough to be added in the latest comprehensive phylogeny of world bumblebees (Cameron et al. 2007) or even barcoded (e.g., all the Afghan *Pyrobombus* and *Psithyrus* species). Uncertainties also remain for the specimens of the cryptic yellow-banded *Bombussensustricto* complex, despite an extensive revision of the subgenus having been performed at the world scale (Williams et al. 2012). Cephalic labial secretions, now studied from dozens of species (e.g., Brasero et al. 2018a, b, Valterová et al. 2020) are totally unknown for all Afghan taxa. Furthermore, while large-scale meta-analyses on numerous bumblebee species have been performed by gathering old and recent material in the Nearctic and West-Palearctic regions (e.g., Kerr et al. 2015; Rasmont et al. 2015), the very scarce Afghan data coming from museum collections and the total absence of recent surveys makes the establishment of such assessments and conservation measures totally impracticable. Collection of fresh material preserved in adequate conditions and more extensive studies of museum collections are therefore essential to fully describe Afghan species and to protect them from the various factors causing declines that impact the genus throughout the world. Above all, we hope that the present study and database will encourage further work on the rich fauna and flora of Afghanistan.

## Supplementary Material

XML Treatment for Bombus (Mendacibombus) makarjini

XML Treatment for Bombus (Mendacibombus) marussinus

XML Treatment for Bombus (Mendacibombus) turkestanicus

XML Treatment for Bombus (Subterraneobombus) melanurus

XML Treatment for Bombus (Psithyrus) branickii

XML Treatment for Bombus (Psithyrus) ferganicus

XML Treatment for Bombus (Pyrobombus) biroi

XML Treatment for Bombus (Pyrobombus) kotzschi

XML Treatment for Bombus (Pyrobombus) subtypicus

XML Treatment for Bombus (Bombus) aff.longipennis

XML Treatment for Bombus (Bombus) tunicatus

XML Treatment for Bombus (Melanobombus) keriensis

XML Treatment for Bombus (Melanobombus) semenovianus

XML Treatment for Bombus (Sibiricobombus) asiaticus

XML Treatment for Bombus (Sibiricobombus) morawitzi

XML Treatment for Bombus (Sibiricobombus) obtusus

XML Treatment for Bombus (Cullumanobombus) cullumanus

## Supplementary materials

Supplementary material 1 Table S1. Bumblebee records examined in the present study Data type species data https://binary.pensoft.net/file/459398Guillaume Ghisbain, Paul H. Williams, Denis Michez, Michael G. Branstetter, Pierre Rasmont
